# An Expeditious Synthesis of *N*-substituted Pyrroles via Microwave-Induced Iodine-Catalyzed Reactions under Solventless Conditions 

**DOI:** 10.3390/molecules15042520

**Published:** 2010-04-09

**Authors:** Debasish Bandyopadhyay, Sanghamitra Mukherjee, Bimal K. Banik

**Affiliations:** Department of Chemistry, The University of Texas-Pan American, 1201 West University Drive, Edinburg, TX 78541, USA

**Keywords:** pyrrole, molecular iodine, microwave irradiation, solventless reaction

## Abstract

An expeditious synthesis of *N*-substituted pyrroles has been developed by reacting 2,5-dimethoxy tetrahydrofuran and several amines using a microwave-induced molecular iodine-catalyzed reaction under solventless conditions.

## 1. Introduction

**Scheme 1:**
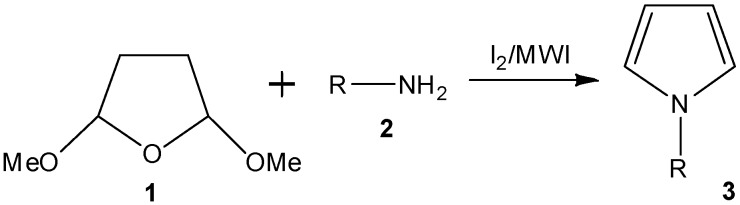
Iodine-catalyzed microwave-induced synthesis of *N*-substituted pyrroles.

Pyrroles are an important class of organic compounds with different types of medicinal activities [1,2], consequently, many methods for the synthesis of diversely substituted pyrroles have been developed [3]. However, the most reliable method for the synthesis of pyrroles is the Paal-Knorr reaction [4,5]. Because of the biological activities of our polyaromatic compounds [6,7,8,9,10], we became interested in the synthesis of pyrroles bound to the amines of different structures. We describe here a simple method of synthesis of *N*-substituted pyrroles by reacting 2,5-dimethoxytetrahydrofuran (**1**) and various amines in the absence of any solvent in a microwave oven in the presence of catalytic amounts (~5 mol%) of molecular iodine. This reaction produces pyrroles in excellent yield and within a short time under (Scheme 1). 

## 2. Results and Discussion

This reaction has been tested by reacting different types of amines **2** and 2,5-dimethoxy-tetrahydrofuran in a microwave oven in the presence of molecular iodine as a catalyst. The reaction yields the products extremely well in the absence of any solvent. Reaction conditions and the yields of the products are shown in Table 1. Both aliphatic and aromatic amines produce pyrroles in very high yield. Importantly, the reaction produces product with multicyclic aromatic amines. A diamine (entry 9) and a heteropolyaromatic amine (entry 10) required higher temperature and longer reaction times, probably due to the diminished availability of the nitrogen (amine) lone pair.

**Scheme 2:**
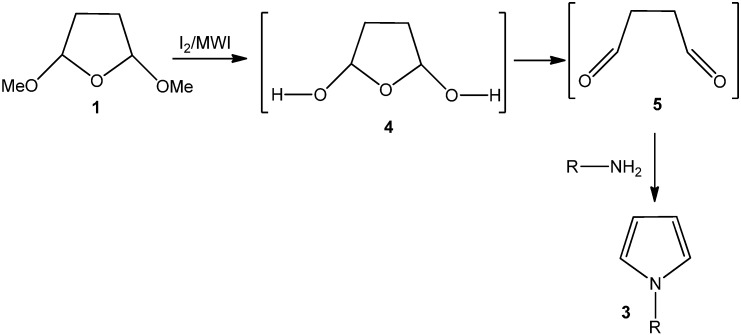
Iodine-catalyzed pyrrole synthesis: plausible mechanism of the reaction.

We hypothesized that our work on iodine-catalyzed [11,12,13,14,15,16] reactions on acetal and glycosylation might prove useful for the facile synthesis of pyrroles under mild conditions. The methoxy groups in 2,5-dimethoxytetrahydrofuran (**1**) can be deprotected under mild acidic conditions and microwave irradiation. The intermediate can easily form the dialdehyde **5**. On reaction with amines and the dialdehyde, pyrroles **3** can be prepared following a nucleophilic addition and subsequent dehydration-aromatization route (Scheme 2). After irradiating a CDCl_3_ solution of **1** for 5 minutes, ^1^H-NMR has been taken. A downfield signal due to the –CHO group is observed. The intensity of the –CHO group becomes more predominant in the ^1^H-NMR when **1** was irradiated in CDCl_3_ in the presence of catalytic amounts of iodine. This suggests the facile formation of dialdehyde **5** in the reaction media in the presence of iodine and microwave irradiation. 

The presence of small amount of iodine (~5 mol %) is essential for the success of the reaction. 

**Table 1:** Microwave-induced iodine-catalyzed synthesis of pyrroles.

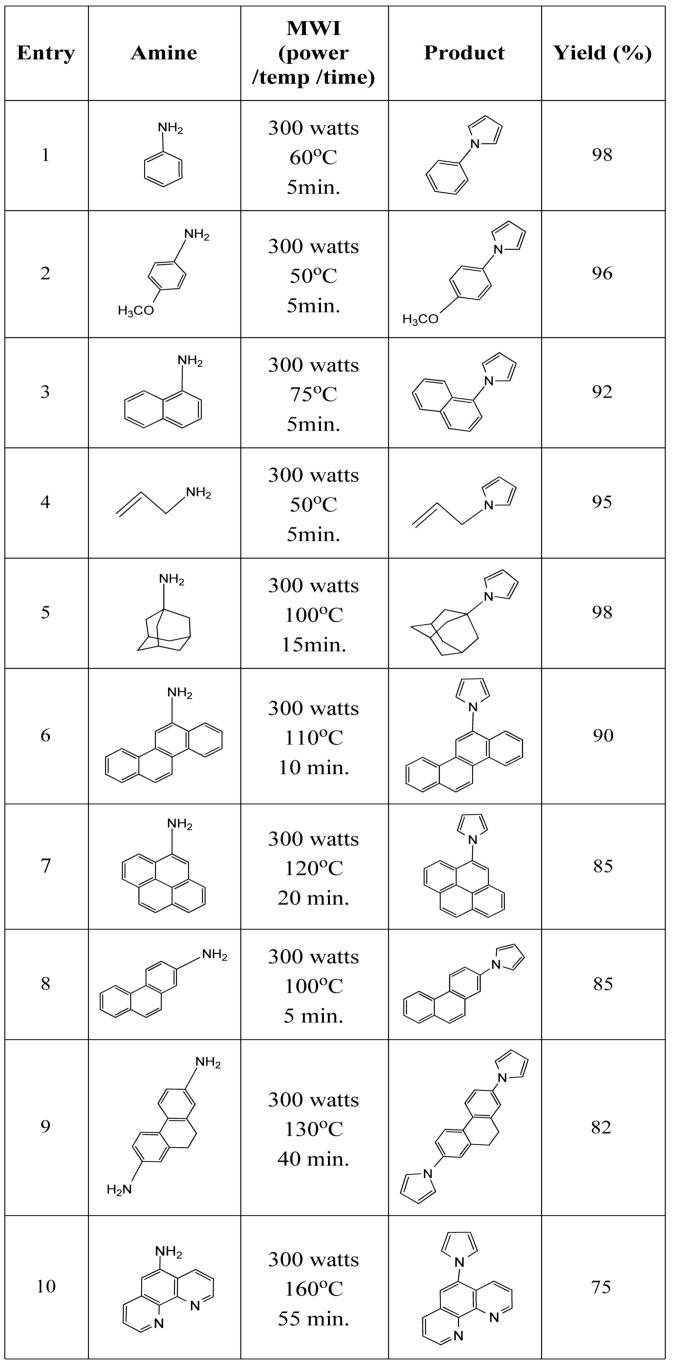

## 3. Experimental

### 3.1. General

Melting points were determined in a Fisher Scientific electrochemical Mel-Temp* manual melting point apparatus (Model 1001) equipped with a 300 °C thermometer. Elemental analyses (C, H, N) were conducted using the Perkin-Elmer 2400 series II elemental analyzer, their results were found to be in good agreement (± 0.2%) with the calculated values for C, H, N. FT-IR spectra were registered on a Bruker IFS 55 Equinox FTIR spectrophotometer as KBr discs. ^1^H-NMR (300 MHz) and ^13^C-NMR (75.4 MHz) spectra were obtained at room temperature with JEOL Eclipse-300 equipment using TMS as internal standard and CDCl_3_ as solvent. Analytical grade chemicals (Sigma-Aldrich Corporation) were used throughout the project. Deionized water was used for the preparation of all aqueous solutions.

### 3.2. General procedure for the synthesis of pyrroles *(**3**)*

Amine **2** (1.0 mmol), 2,5-dimethoxytetrahydrofuran (**1**, 1.2 mmol) and iodine (5 mol%) was irradiated in a CEM automated microwave oven, as specified in Table 1. After completion of the reaction (monitored by TLC) diethyl ether (10 mL) was added to the reaction mixture which was then filtered. Pure product was isolated from the reaction mixture after evaporation of ether. Spectroscopic data for representative compounds, e.g. monoaromatic (entries 1 and 2), alicyclic (entry 5), polyaromatic (entries 6 and 7) as well as heteropolyaromatic (entry 10) are as follows: 

*1-Phenyl-1H-pyrrole* (**3a**, entry 1). Brown sticky oil. IR: 2923, 1312, 1106, 782, 611 cm^-1^; ^1^H-NMR δ (ppm): 6.39 (m, 2H, pyrrole), 7.24 (d, 2H, *J = 3.12*, pyrrole), 7.42–7.53 (m, 5H, Ar-H); ^13^C-NMR δ (ppm): 109.07 (2C), 115.51 (2C), 122.96 (2C), 124.57, 128.62 (2C), 137.08. Anal. Calcd. for C_10_H_9_N: C, 83.88; H, 6.34; N, 9.78. Experimental: C, 83.79; H, 6.31; N, 8.72.

*1-(4-Methoxyphenyl)-1H-pyrrole* (**3b**, entry 2). Black amorphous solid. Solidified from dichloromethane/hexane mixture; Mp: 89 °C; IR: 2954, 1301, 1191, 850, 748 cm^-1^; ^1^H-NMR δ (ppm): 3.87 (s, 3H, OCH_3_), 6.33 (m, 2H, pyrrole), 6.67 (d, 2H, *J* = 3.30), 6.96–7.73(m, 4H, Ar-H); ^13^C-NMR δ (ppm): 55.66 (OCH_3_), 109.93 (2C), 114.71 (2C), 120.15 (2C), 126.07 (2C), 132.56, 158.21. Anal. Calcd for C_11_H_11_NO: C, 76.28; H, 6.40; N, 8.09. Experimental: C, 76.20; H, 6.27; N, 7.97.

*1-(Adamntan-1-yl)-1H-pyrrole* (**3e**, entry 5). Yellow crystals. Crystallized from diethyl ether/hexane mixture; Mp: 71 °C; IR: 2923, 2855, 1479, 1450, 1219, 713, 619 cm^-1^; ^1^H-NMR δ (ppm): 1.77 (m, 3H), 2.12 (d, 6H, *J* = 3.00), 2.23 (m, 6H), 6.19 (t, 2H, *J* = 2.19, pyrrole), 6.91(m, 2H, pyrrole); ^13^C-NMR δ (ppm): 29.79 (3C), 36.35 (3C), 44.04 (3C), 55.00, 107.24 (2C), 116.55 (2C). Anal. Calcd for C_14_H_19_N: C, 83.53; H, 9.51; N, 6.96. Experimental: C, 83.41; H, 9.50; N, 6.89.

*1-(Chrysen-6-yl)-1H-pyrrole* (**3f**, entry 6). Brown crystals. Crystallized from ethyl acetate/hexane mixture; Mp: 139 °C; IR: 2947, 2924, 1513, 1461, 1071, 812 cm^-1^; ^1^H-NMR δ (ppm): 6.49 (t, 2H, *J* = 2.21, pyrrole), 7.12 (m, 2H, pyrrole), 7.70–8.72 (m, 11H, Ar-H); ^13^C-NMR δ (ppm): 109.27 (2C), 120.98, 121.29 (2C), 123.23, 123.49 (2C), 123.64 (2C), 124.05, 126.45 (2C), 126.75 (2C), 126.88 (2C), 127.42, 128.64 (2C), 132.26, 137.94. Anal. Calcd for C_22_H_15_N: C, 90.08; H, 5.15; N, 4.77. Experimental: C, 89.91; H, 5.04; N, 4.76.

*1-(Pyren-1-yl)-1H-pyrrole* (**3g**, entry 7). Pale yellow crystals. Crystallized from diethyl ether/hexane mixture; Mp: 78 °C; IR: 2956, 2927,1598, 1511, 1304, 1072, 848 cm^-1^; ^1^H-NMR δ (ppm): 6.53 (m, 2H, pyrrole), 7.15 (m, 2H, pyrrole), 8.01–8.19 (m, 9H, Ar-H); ^13^C-NMR δ (ppm): 109.45 (2C), 122.36, 123.83 (2C), 124.89, 125.42, 126.51, 126.72, 127.19, 127.89 (2C), 128.63 (2C), 128.92, 130.62, 131.01, 131.39, 133.43, 135.97. Anal. Calcd for C_20_H_13_N: C, 89.86; H, 4.90; N, 5.24. Experimental: C, 89.77; H, 4.81; N, 5.17.

*5-(1H-Pyrrol-1-yl)-1,10-phenanthroline* (**3j**, entry 10). Yellowish brown amorphous solid. Solidified from methanol/hexane mixture; Mp: 192 °C; IR: 3186, 2362, 2336, 1591, 1539, 1074, 739 cm^-1^; ^1^H-NMR δ (ppm): 6.44 (broad s, 2H, pyrrole), 7.02 (broad s, 2H, pyrrole), 7.62–9.20 (m, 7H, Ar-H); ^13^C-NMR δ (ppm): 110.14 (2C), 122.79, 123.24 (2C), 123.55 (2C), 123.74 (2C), 129.04, 132.26, 136.17, 143.91, 144.09, 150.80, 150.91. Anal. Calcd for C_16_H_11_N_3_: C, 78.35; H, 4.52; N, 17.13. Experimental: C, 78.23; H, 4.43; N, 17.02.

## 4. Conclusions

In summary, the present iodine-catalyzed microwave-induced method in the absence of any solvent is excellent for the preparation of *N*-substituted pyrroles (75–98% yields). Because of the simplicity of the procedure, products can be isolated very easily. The compounds reported herein will be tested against a number of cancer cells *in vitro*.
